# Visual Genomics Analysis Studio as a Tool to Analyze Multiomic Data

**DOI:** 10.3389/fgene.2021.642012

**Published:** 2021-06-17

**Authors:** Rebecca J. Hertzman, Pooja Deshpande, Shay Leary, Yueran Li, Ramesh Ram, Abha Chopra, Don Cooper, Mark Watson, Amy M. Palubinsky, Simon Mallal, Andrew Gibson, Elizabeth J. Phillips

**Affiliations:** ^1^Institute for Immunology and Infectious Diseases, Murdoch University, Murdoch, WA, Australia; ^2^Department of Medicine, Vanderbilt University Medical Centre, Nashville, TN, United States

**Keywords:** bioinformatics, heterogeneity, immunogenomics, single-cell TCR sequencing, single-cell RNA sequencing, single-cell CITE-seq

## Abstract

Type B adverse drug reactions (ADRs) are iatrogenic immune-mediated syndromes with mechanistic etiologies that remain incompletely understood. Some of the most severe ADRs, including delayed drug hypersensitivity reactions, are T-cell mediated, restricted by specific human leukocyte antigen risk alleles and sometimes by public or oligoclonal T-cell receptors (TCRs), central to the immunopathogenesis of tissue-damaging response. However, the specific cellular signatures of effector, regulatory, and accessory immune populations that mediate disease, define reaction phenotype, and determine severity have not been defined. Recent development of single-cell platforms bringing together advances in genomics and immunology provides the tools to simultaneously examine the full transcriptome, TCRs, and surface protein markers of highly heterogeneous immune cell populations at the site of the pathological response at a single-cell level. However, the requirement for advanced bioinformatics expertise and computational hardware and software has often limited the ability of investigators with the understanding of diseases and biological models to exploit these new approaches. Here we describe the features and use of a state-of-the-art, fully integrated application for analysis and visualization of multiomic single-cell data called Visual Genomics Analysis Studio (VGAS). This unique user-friendly, Windows-based graphical user interface is specifically designed to enable investigators to interrogate their own data. While VGAS also includes tools for sequence alignment and identification of associations with host or organism genetic polymorphisms, in this review we focus on its application for analysis of single-cell TCR–RNA–Cellular Indexing of Transcriptomes and Epitopes by Sequencing (CITE)-seq, enabling holistic cellular characterization by unbiased transcriptome and select surface proteome. Critically, VGAS does not require user-directed coding or access to high-performance computers, instead incorporating performance-optimized hidden code to provide application-based fast and intuitive tools for data analyses and production of high-resolution publication-ready graphics on standard specification laptops. Specifically, it allows analyses of comprehensive single-cell TCR sequencing (scTCR-seq) data, detailing (i) functional pairings of α–β heterodimer TCRs, (ii) one-click histograms to display entropy and gene rearrangements, and (iii) Circos and Sankey plots to visualize clonality and dominance. For unbiased single-cell RNA sequencing (scRNA-seq) analyses, users extract cell transcriptome signatures according to global structure via principal component analysis, t-distributed stochastic neighborhood embedding, or uniform manifold approximation and projection plots, with overlay of scTCR-seq enabling identification and selection of the immunodominant TCR-expressing populations. Further integration with similar sequence-based detection of surface protein markers using oligo-labeled antibodies (CITE-seq) provides comparative understanding of surface protein expression, with differential gene or protein analyses visualized using volcano plot or heatmap functions. These data can be compared to reference cell atlases or suitable controls to reveal discrete disease-specific subsets, from epithelial to tissue-resident memory T-cells, and activation status, from senescence through exhaustion, with more finite transcript expression displayed as violin and box plots. Importantly, guided tutorial videos are available, as are regular application updates based on the latest advances in bioinformatics and user feedback.

## Introduction to Visual Genomics Analysis Studio: Analysis and Visualization of Multidimensional Single-Cell Sequencing Data

Understanding the immunogenic risk factors associated with immune-mediated disease has seen significant progress in recent decades, linked to the rapid and continued development of genomic sequence–based technologies initially at a bulk and more recently at a single-cell level. While progress spans an entire spectrum of immune disease, T-cell–mediated delayed drug hypersensitivity reactions (DHRs) stand out as some of the strongest associations with distinct human leukocyte antigen (HLA) alleles. These HLA associations are specific to reaction phenotype, drug, and patient ethnicity (Chung et al., 2004; Lonjou et al., 2006; Wu et al., 2016; Nakkam et al., 2018; Konvinse et al., 2019). However, for all associations to date, a positive predictive gap remains (Phillips, 2018), limiting the clinical impact of HLA screening for particular drugs and our understanding of reaction mechanisms, driving active research to identify those additional risk parameters imposed on HLA-restricted response. In 2019, a role for specific T-cell receptor (TCR) clonotypes among the expansive human repertoire was reported by Pan et al., who detailed expression of a single, dominant, public TCR in the reacted skin of patients with HLA-B^∗^15:02-restricted carbamazepine Stevens–Johnson syndrome and toxic epidermal necrolysis (SJS/TEN), but which was absent from tolerant controls and healthy donors (Pan et al., 2019). Using genetic engineering to insert a synthetic construct of the dominant TCR into a murine drug-exposure model, they showed that this TCR recognized carbamazepine, functionally validating the risk TCR as a key driver of early drug-specific response in tissue. Aside from inferring critical structural restriction regarding binding of the drug and/or peptide by the HLA and TCR molecules to bridge the immunological synapse, the dominantly expanded TCR may serve as a functional biomarker to identify and characterize the specific effector populations driving disease. Cost-effective genetic screening pipelines for HLA and other polymorphic genes see continued clinical progress toward better genetic risk prediction (Rauch et al., 2006; Chen et al., 2011; Plumpton et al., 2015). However, mechanistic understanding of these reactions has been hampered by limited availability of singular platforms for fully integrated user-friendly analyses by the non-coding-proficient researcher. Techniques including flow cytometry, microscopy, *in situ* hybridization, and more recently mass cytometry have been utilized, yielding insights into the phenotype of cells participating in response, but have not simultaneously characterized the TCRs and transcriptome and surface protein markers at a single-cell level (Chattopadhyay et al., 2014). Comparatively, sequencing-based strategies have delivered unrivaled opportunity as markers are tagged with synthetic DNA barcodes, providing truly limitless sequence combinations for high-dimensional detection of RNA or protein, or indeed distinct pooled (“hashed”) samples to enhance cost efficiency per run. These techniques were initially applied to bulk analyses, providing average expression across an entire sample and so potentially hiding response from individual effector populations preventing resolution from total sample expression. Thus, as with all bulk assays, opportunity to detail the true complex cellular heterogeneity of clinical samples was lost, but which is integral to complete understanding of disease as immune interactions are complex and continuously regulated by intercell interactions and secretions. Concurrent with advances in cell sorting and droplet technologies, single-cell sequencing by Smart-Seq2 and 10x platforms, respectively, now provide information for each and every cell (Nguyen et al., 2018; Svensson et al., 2018). With the support of bioinformatics-driven algorithms, the complete transcriptomic signature of each cell provides means to cluster similar cells without user-directed imposition of preconceived expression, which, when aligned to the open-access human cell atlas under continued development, enables verification of subsets identified through unsupervised clustering for user-directed signature analyses. Already this technology is revealing a spectrum of heterogeneous clusters within previously thought homogeneous populations (Villani et al., 2017), driving immunogenic discovery across the spectrum of immune-mediated diseases. These platforms now present as fully integrated, multifocal pipelines for simultaneous assessment of (i) unbiased transcriptome (single-cell RNA sequencing; scRNA-seq), (ii) select surface proteome (single-cell Cellular Indexing of Transcriptomes and Epitopes by Sequencing; scCITE-seq), and, for T-cells, (iii) functional TCR (single-cell TCR sequencing; scTCR-seq) α–β pairings with VDJ complementarity determining region 3 (CDR3) inference of antigen restriction. Importantly, aside from discovery analyses via differential expression, investigational studies can be performed on interest markers in distinct subpopulations with exquisite specificity. Specific allelic risk variants on interacting cells can also be identified and investigated, which is important given the observation that the level of HLA expression, beyond a simple yes or no presence, impacts effector response (Thananchai et al., 2007). Critically, samples pooled using hashtags within a single analysis for overlaid visual inspection or differential expression provide opportunity to detail similarities or discrepancies between samples with unique clinical metadata such as disease phenotype or mortality. These approaches provide an opportunity to discover disease-specific cell populations and targets for development of diagnostic tools or treatments.

The advent of microfluidics devices to accurately encapsulate single cells in droplet suspension, barcoding, and contemporary sequencing now delivers high-yield single-cell data. Consequently, the needs have moved to data quality assurance, management (informatics), analysis, and visualization, specifically, how to qualify and interpret the immense amount of complex data acquired. This traditionally necessitates specialist bioinformatics support, coding expertise, and access to high-end and robust computing software and servers, imposing a significant cost of implementation to laboratories of all sizes (Lähnemann et al., 2020). While data analysis tools are typically developed at pace with advancements in sequencing technologies, they are mostly limited to command line usage of code, restricting their direct utility to research scientists and clinicians. Moreover, although other platforms exist for similar analyses, many are designed for singular and specific utilities. To navigate, we present Visual Genomics Analysis Studio (VGAS), a Windows-aligned, application-based intuitive graphical user interface with performance-optimized hidden code to drive comprehensive differential analyses, specifically designed to allow basic and clinical researchers to interrogate and dissect their own data and generate publication-ready visuals for presentation on standard specification laptops. It is designed to package existing tools in a single accessible format, otherwise beyond the reach of basic researchers lacking coding proficiencies for a variety of analysis options. Moreover, VGAS, which also incorporates tools for sequence alignment and viral integration site analyses, remains constantly updated to incorporate user-defined features and recently developed tools from this rapidly evolving field. Here, with reference to screenshots and web-accessible tutorial videos, we introduce the investigation capacity offered by single-cell analyses in the context of VGAS. Critically, while our own translation discussed within is tailored to focus on T-cell–mediated DHRs, the functions offered are broadly applicable to single-cell study of diverse samples and diseases. Here we demonstrate the utility of VGAS using a 10x Genomics test dataset (available at: https://support.10xgenomics.com/single-cell-vdj/datasets/5.0.0/sc5p_v2_hs_PBMC_10k_multi_5gex_5fb_b_t) in a VGAS-compatible matrix file (available at https://www.iiid.com.au/software/vgas).

### Utility of scTCR-Seq: Diversity and Dominance Define Tissue and Antigen Restriction

Within each individual, there is enormous diversity in TCRα–β heterodimer repertoire, with an estimated 10^13^–10^16^ unique TCR per person (Robins et al., 2009; Soto et al., 2020). In T-cell–mediated DHRs, where specific HLA alleles expressed on antigen-presenting cells are associated with immunogenic risk, distinct TCRs have recently been reported to similarly restrict response on the corresponding effector T-cells (Pan et al., 2019). Specifically, through use of scTCR-seq, capable of ascertaining α–β TCR pairs, Pan et al. identified a single, public TCRβ CDR3 “ASSLAGELF” paired with TCRα CDR3 “VFDNTDKLI” dominantly expanded in the blister fluid of multiple patients with HLA-B^∗^15:02-restricted carbamazepine-induced SJS/TEN. Importantly, this pairing was absent from healthy and tolerant controls, and while present in peripheral blood mononuclear cells from the same allergic patients, abundance was far lower than detected in blister fluid. Such TCR specificity is similarly identified for other immunodominant T-cell responses in alternate disease settings, including infectious disease, such as inflated HLA-B^∗^44:03-restricted CMV-specific CD8^+^ T-cell responses to a defined immunodominant immediate-early 2 derived epitope (Attaf et al., 2018). Importantly, TCR specificity may also provide an explanation for the skin-directed targeting of cutaneous DHR by more widely distributed drugs, as the T-cell tissue-resident repertoire is not consistent throughout the body but compartmentalized into enclaves specific to tissue, in part directed by previous antigenic exposures (Kumar et al., 2018). Indeed, large populations of antigen-specific tissue-resident memory (Trm) effector T-cells reside within organs, with those in the skin and mucous membranes, typical microbial entry sites, distributed as such for rapid activation following secondary exposures. These microbially primed Trm cells are retained in the skin but remain motile within, with recently demonstrated capacity to proliferate (Behr et al., 2018). Such tissue-specific locality of viral-specific T-cell effectors with epitope-restricted TCR reactivity is the basis of the heterologous immunity model, whereby drug antigens may cross-react with viral-specific T-cells, driving the tissue-specific targeting of these reactions (Pavlos et al., 2015). To this end, even simple comparative analysis of TCR repertoire between affected and unaffected sample may be informative as divergent expression would be indicative of an active immune infiltrate, or conversely, similarity may point to activation of tissue-retained effectors. These considerations emphasize (a) the importance of collecting clinically relevant tissue samples during early response to detect drug- and disease-specific dominant TCR expansion and (b) the utility of scTCR-seq to detail such dominance and provide α–β (and J chain) structure of the TCR for further functional studies. Indeed, while traditional TCR analyses have focused on expression of the β chain alone, including CDR3 spectra typing and flow cytometry–based detection kits, the influence of the corresponding TCRα variable (TRAV) chain and associated CDR3 to define antigen specificity is critical (Gras et al., 2008; Rossjohn et al., 2015). Thus, scTCR-seq provides complete human paired TCRα–β sequences enabling synthetic reconstruction for functional validation with culprit antigen and risk HLA as demonstrated by Pan et al. using an engineered murine model (Pan et al., 2019).

### VGAS: scTCR Analyses and Visualization Tools

**FIGURE 1:**
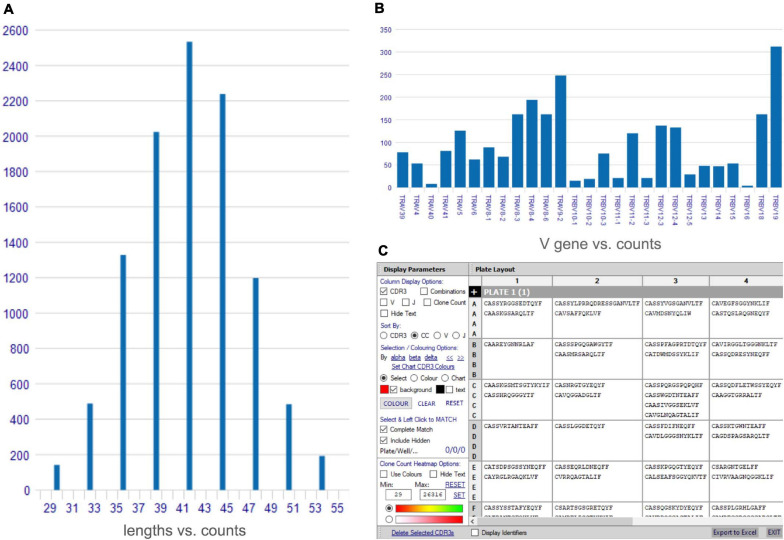
Access tools for analysis of single-cell TCR-seq data. A variety of rapid access tools are available for analyses of single-cell TCR-seq data including **(A)** comparative CDR3 length histogram, **(B)** gene frequency histogram for individual TCR genes (TRV α and β genes shown), **(C)** plate/well layout explorer for plate-based assays listing paired CDR3 chain sequence, with options to show individual genes.

VGAS provides a platform for multidimensional analysis of scTCR-seq with tools to visualize dominance of α–β combinations and respective CDR3 sequences^1^ (TCR analysis tutorials). Several one-click functions are available direct from the scTCR-seq home menu screen after file upload, including comparative graphical presentation of CDR3 lengths (see *TCR analysis tutorials: CDR3 length*) (Figure 1A), scatterplots to represent TCRs common to samples (see *TCR analysis tutorials: scatterplot*), and VDJ gene frequency histograms (see *TCR analysis tutorials: gene frequency*) (Figure 1B) in linear or logarithmic scaling with filters to exclude non-productive TCR containing stop codons or out-of-frame alignments. Further, the “CDR3/well plate explorer” function (Figure 1C) provides the full detailed numerical list view of TCR pair representation in each well to link with multimodal or functional data if performing plate-based assays (Smart-Seq2).

**FIGURE 2:**
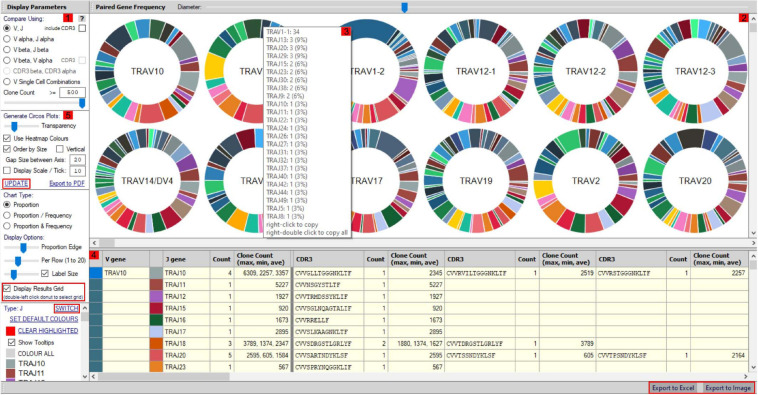
scTCR-seq paired gene/CDR3 frequency window. The scTCR-seq analysis home screen window is divided into key domains to help direct analyses by the user. **(1)** Gene pairings or corresponding CDR3 sequences can be selected for analysis by the user within the “display parameters” domain. **(2)** Representative pie charts display proportion (and frequency) of TCR genes/CDR3 within the selected sample for initial indication of data. Each pie depicts one TCR chain with the colored border representing proportional number of pairings with corresponding chains as selected in domain 1. The central chain can be switched using “Switch” (highlighted red) in the parameters panel for alternate pairing view. **(3)** Hovering over select pies provides an information box with proportional representation (%) of total pairings within. **(4)** Paired genes are displayed in grid format by selecting “display results grid” (highlighted red), which can be exported direct to Excel or Image (highlighted red). **(5)** Circos plots can be generated for selected samples/pairings by clicking “Update” (highlighted red).

**FIGURE 3:**
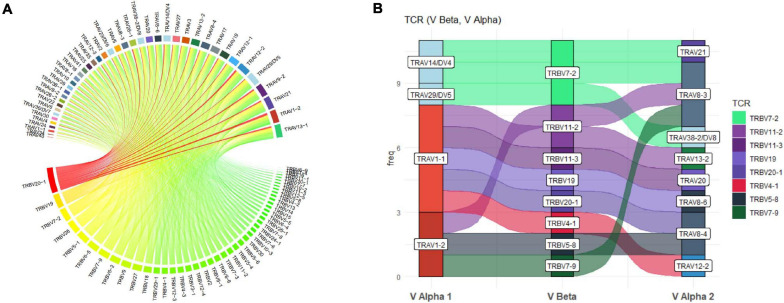
Circos and Sankey plots to display holistic clonality, dominance, and complex pairings. **(A)** Circos plots provide an overview of TCR pairing clonality and dominance within a selected sample as shown for TRAV and TRBV pairings and can also be produced for paired CDR3. Alpha genes are listed at the top and paired to respective β genes at the bottom with width and color (red to green) of each segment proportionate to comparative dominance of total functional TCR pairings. **(B)** Sankey plot illustrating more complex gene interactions identified by sequencing, shown for TRAV-TRBV-TRAV triad, detailing clone frequency on *y*-axis.

More detailed visualization of dominance and holistic α–β TCR pairings to detail clonality within and between samples is possible via the main TCR analysis screen (Figure 2). Users can select individual pairings of interest including α and β chains or α and β CDR3 (Figure 2(1)). This selection is visualized in the “paired gene frequency” domain (Figure 2(2)), where one chain is depicted per pie and the colored edge surrounding indicative of the number and proportion of pairings with alternate corresponding chains. An active cursor hover tool over each pie provides full details of each pairing and comparative percentage expression (Figure 2(3)), with full details of all pairings, inclusive of J chains and CDR3 observed in the bottom window (Figure 2(4)) from which BLOSUM scoring can ascertain sequence similarity for inference of similar restriction between different TCRs. From here, complex TCR visuals can be generated using the “Generate Circos plots” domain, specifically through selection of the “Update” toggle (Figure 2(5)). Circos plots, linking one chain at the top to another at the bottom, are best suited for holistic representation of clonality and are auto colored to indicate dominant (red) compared to subdominant (green) TCRs. The extent of a specific pairing between an α and β chain is represented by the width of each connection (Figure 3A). The settings for these Circos plots are configurable. For example, plots may be set to visualize the α and β combinations but also α or β and respective J chains, or α and β CDR3 sequences, which ultimately define peptide specificity. For ease of identification, each segment is annotated in the plot, which can be set to display proportion and/or frequency of combinations. Scaled plots may be exported to an image or raw data exported to Excel to produce tables and access numerical representation of TCR within. Sankey plots provide an alternate view for complex pairing interactions between single α chains and multiple β chains or *vice versa* (Figure 3B). This is often appropriate for a restricted data set, i.e., top 20 pairings. Each chain can be moved independently up or down the figure such that a TCR of interest can be listed as required. Sankey plots therefore allow visualization of pairings between two α genes or two β genes as are increasingly being detected by single-cell sequencing. A new feature also allows the TCR repertoire for a specific study to be instantly compared to that of previous samples, stored as an active database, which can be adapted for inclusion of external datasets, such that the user can search for similar findings in other studies.

### Utility of scRNA-Seq: Unsupervised Holistic Dissection of Signature Transcriptomes

For certain reactions such as abacavir hypersensitivity, TCR responses appear to be polyclonal, suggesting a role for diverse immunogenic antigens, and epitopes in keeping with the altered peptide hypothesis (Redwood et al., 2019). However, as described above for carbamazepine, single dominant TCRs have been identified in patient blister during reaction, providing an antigen-relevant functional marker to identify and characterize the critical effector population driving destructive disease. While output cellular functionality is largely imposed by surface protein, the transcriptome is the vastly more complex precursor, now almost completely measurable by scRNA-seq without bias of preselect markers providing a holistic transcriptomic signature for an individual cell. Algorithms, outlined by Allaoui et al. (2020), then “pull” similar RNA signatures together by k-means clustering, enabling visualized clusters in t-distributed stochastic neighborhood embedding (t-SNE) or uniform manifold approximation and projection (UMAP) plots to be grouped and independently characterized direct from sample suspension.

Unbiased transcriptome analyses have recently proven utility in defining critical cellular signatures with influence in varied diseases including cancer (Dai et al., 2019; Zhang et al., 2020) and infection (Bossel Ben-Moshe et al., 2019; Yao et al., 2019), driving immunological understanding and identification of disease-relevant biomarkers (Szabo et al., 2019; Cheng et al., 2020). Similar application to DHRs remains limited to a handful of studies, one detailing rechallenge response during HLA-restricted positive patch test (Redwood et al., 2019), and another the effector signature during a single case of treatment-refractory drug reaction with eosinophilia and systemic symptoms (Kim et al., 2020). In this latter study, the merit of unbiased scRNA-seq to directly identify targetable biomarkers of disease was clearly demonstrated when investigators found the JAK-STAT pathway to be enriched in effectors directing the clinical investigators to repurpose tofacitinib and effectively control disease (Chattopadhyay et al., 2014). Application across samples from patients with similar reactions may therefore provide more distinct, reaction-specific biomarkers. However, scRNA-seq captures the transcriptome at unparalleled resolution, posing a challenge for managing, analyzing, and visualizing data. While a range of software has been developed within tools such as R for the analysis of high-dimensional datasets, the user must be proficient in this type of programming and its strict framework, reducing capacity for the researcher to freely explore their data.

### VGAS: scRNA Analyses and Visualization Tools

**FIGURE 4:**
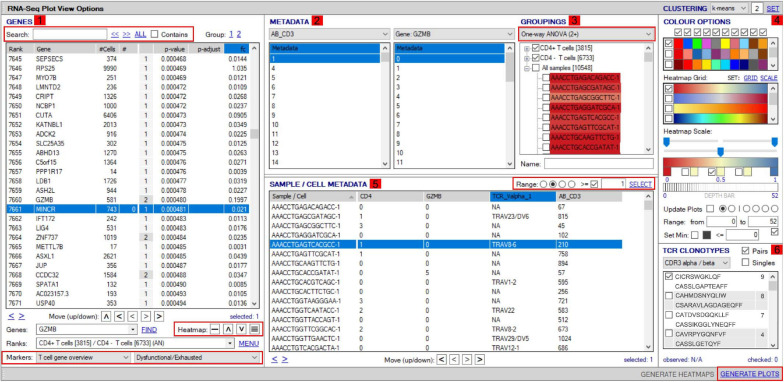
Workflow for analyses of scTCR–RNA-seq data using VGAS plot viewer window. The scTCR–RNA-seq analysis home screen window is divided into key domains to help direct analyses by the user. **(1)** “Genes” panel lists all genes expressed with options to search for specific genes of interest (highlighted red), reorder, and find literature determined groups of interest genes using the “Markers” dropdown tab or heatmap (highlighted red) individual genes on the UMAP for selected sample. **(2)** “Metadata” is displayed with dropdown tabs to select data by sample, clinical metadata, or phenotype groupings defined by indexed flow data or via unsupervised K means clustering on whole sample with consensus calling from reference atlas databases. **(3)** Grouped data/clusters appear in the “Groupings” domain for selection and differential analyses using a range of statistical algorithms contained in the dropdown tab (highlighted red). Differential gene analysis results provide *p*-value, *p*-adjusted, and fold change displayed in “Genes” panel. **(4)** Groups and plots can be fully color-customized in the “Color options” panel and heatmap scales set to user preference. **(5)** Genes of interest, metadata, or groupings can be saved and set as metadata for further interrogation or grouping by several parameters in the “Sample/cell metadata panel.” User-defined positive cutoff range for numerical data can be applied to filter samples using “Range” function toggle (highlighted red). **(6)** scTCR-seq defined clonotypes can be set from whole data or distinct RNA-defined groups in the “TCR clonotypes” domain. Circos plots can also be generated from within this panel. The “generate plots” function, bottom right (highlighted red), provides access to associated UMAP.

VGAS integrates a number of genomic dataset tools developed for specific scRNA-seq analyses^2^ (RNA-seq plot viewer tutorials). These include k-means clustering and UMAP/t-SNE plots, varied differential expression analyses with statistical inference, and plots to compare expression levels of specific transcripts. Although suited to both UMAP and t-SNE, VGAS typically uses UMAP, a well-documented non-linear dimensionality reduction algorithm to convert high-dimensional scRNA-seq data into a visual representation maintaining the global structure of data. Compared to alternative t-SNE algorithms, UMAP presents a faster method of clustering with reproducibility and conservation of subtle differences in cellular populations (Becht et al., 2019). Typically for data generated at our center, a single VGAS plot view (VGAS.pv) file is released to the end-user after bioinformatics quality control, for simple upload to VGAS, first opening the single plot viewer control screen from which all analyses are performed (Figure 4). The file is inclusive of all batched samples in a combined UMAP; however, the master normalized gene expression count (.csv) and metadata (.txt) files are additionally provided should the user choose to recapitulate the UMAP modeling. This can be directed through VGAS with automated R plot functions for generation of typical Euclidean, Manhattan, Cosine, Pearson, and Pearson 2 distributions. All distributions may be selected, and plots toggled between as required in the “Plot Viewer.”

**FIGURE 5:**
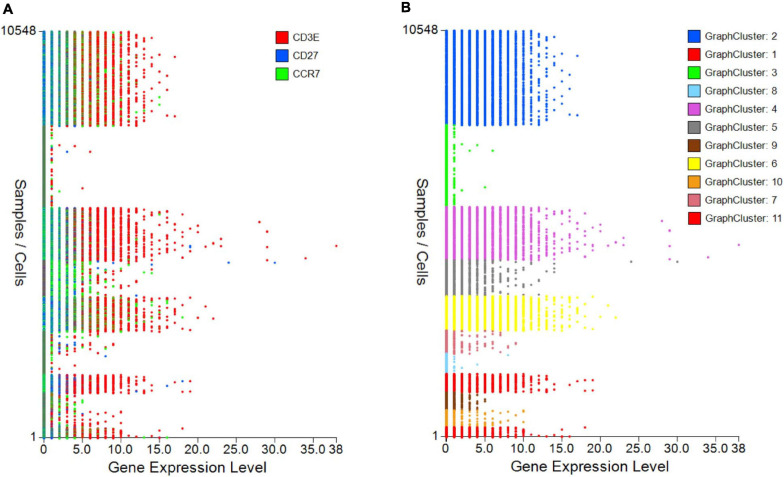
Gene expression plots to provide cutoffs for negative/positive expression or show differences between samples/groups. **(A)** Single or grouped gene expression plots for all cells within a selected sample provides a platform to set cutoff for positivity and identify outlier expression or **(B)** highlight expression differences between samples/plates.

The control “Plot viewer” screen is split into six key domains: “Genes,” “Metadata,” “Groupings,” “Sample/cell metadata,” “Color options,” and “TCR clonotypes,” respectively, depicted in Figure 4(1–6). All metadata are accessible in the control panel “Metadata” domain, e.g., patient ID, sex, age, etc., dependent on that provided by the investigator, offering flexibility and customizability to analyses (Figure 4(2)). The same “Metadata” dropdown menu also holds information acquired during the initial bioinformatics quality assurance and UMAP generation, including cell cycle phase and assigned cell clusters according to individual human cell atlases from which consensus is drawn. This provides the user with an initial overview of the populations present, which can then be highlighted on the UMAP (see *RNA-seq plot viewer tutorials: coloring plots by metadata*) or grouped with ease through one-click selection from the right click control panel and which then appear in the “Groupings” domain (see *RNA-seq plot viewer tutorials: creating groups*) (Figure 4(3)). Of note, each group may be formatted by color, which will translate to all subsequent visuals, through the “Color options” domain (Figure 4(4)). More specific groupings according to select gene expression (all genes listed in the left hand “Genes” domain (Figure 4(1)) can then be developed through import to the “Sample/cell metadata” domain (Figure 4(5)) by selecting either a preselected “Metadata” or individual gene and selecting “Set as metadata.” Four metadata columns are available for combined parameter assessment; however, sequential groupings and reapplication as metadata allow limitless combinations to build a single signature. Imported RNA data are numerical, and the user must define cutoffs for positivity/negativity before analysis. This is possible through the single gene expression plot function to graph the spectrum of gene expression from low to high, which, in a manner similar to conventional histogram-based gating in flow cytometry, provides the user guidance of where to set the positive gate and may further identify bimodal or multimodal expression within or between samples for further gating or low, mid, or high expressors (Figure 5) (see *RNA-seq plot viewer tutorials: generating gene expression level plots*). When defined, the boundary value above a deemed positive can be input into the “Range” function at the top of the “Sample/cell metadata” domain, stripping those not meeting this inclusion criteria from selection in the sample grid. The TCR repertoire for each group, ascertained using the right-click function to generate the correlating data in the “TCR clonotypes” domain (Figure 4(6)), can be used to directly generate Circos plots.

**FIGURE 6:**
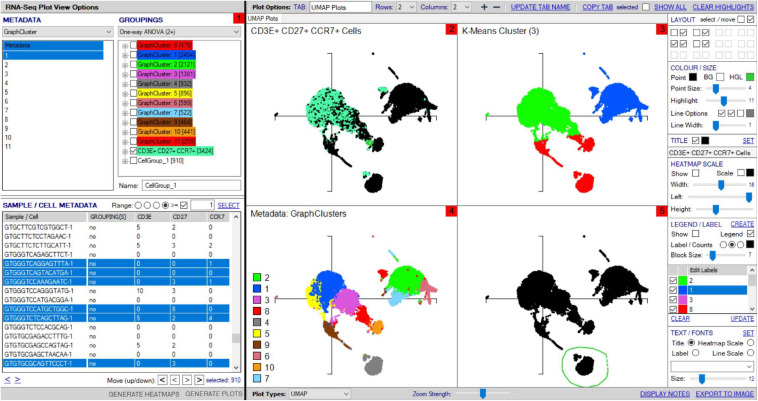
Multipanel UMAP investigation. **(1)** Sample/cell groupings created in “Groupings” panel can be plotted on multipanel UMAPs, with zoomed view possible to focus on different regions of interest (2–5). **(2)** Groupings created from differentially expressed genes and metadata can be highlighted within the total cell population, shown for CD3E^+^CD27^+^CCR7^+^ cells group defined in panel 1. **(3)** K-means clustering can be set in the scRNA-seq Plot View Options window and illustrated within UMAP. **(4)** Cell types from unsupervised clustering can be separately colored on a UMAP with legend for visualization. **(5)** Cell clusters can be “lassoed” on the UMAP and highlighted within the “Sample/cell metadata” grid for addition to distinct groups within “Groupings” for further analyses, named “CellGroup_1” in groupings tab.

Each group can be renamed and identified using the highlight tool by opening a UMAP plot through the “Generate plots” function, which has fully scalable *x* and *y* axes and formatting functions for coloring, titles, and sizing. Multipanel UMAPs can be generated in the plot viewer for easy comparison between cell populations (Figure 6(1–5)). As alternative to groupings defined by metadata (Figure 6(2–4)), groups can also be selected directly via the UMAP using the highlight function in the plot viewer to lasso an interest population before highlighting and grouping these cells within the sample grid (Figure 6(5)). Such analysis may be particularly useful for subclustering islands that fall within the same cell-type metadata consensus, i.e., CD8^+^ T-cells that express dominant-TCR versus non-dominant TCR, for authentic signature exploration.

**FIGURE 7:**
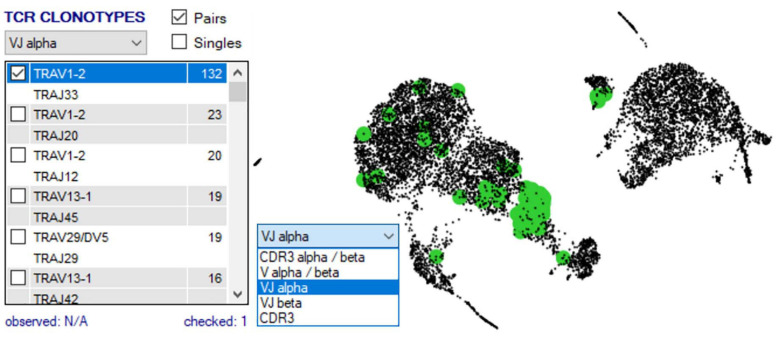
scRNA-defined UMAP overlay of scTCR-seq. Single-cell defined functional TCR clonotypes can be imported for selected populations/samples, displayed in order of dominance within the Plot View Options window, and highlighted on the scRNA-defined UMAP to pinpoint clusters with expression of dominant or interest TCR (highlighted green).

Once two or more groups are created and selected in the “Groupings” domain, the number of cells in each group is shown in brackets, and then two analyses are typically done. First, TCR clonotypes in a particular group can be set via the right-click menu to appear in the bottom right “TCR clonotype” domain if scTCR–RNA-seq was performed, listing combinations of α–β pairs or respective CDR3 pairs in order of dominance (Figure 7). Of note, if scTCR-seq was not performed but a defined interest TCR is known, gene selection for individual TRAV and TRBV within the “Genes” panel will provide an indication of the transcriptome of cells expressing those TRAV or TRBV genes. Selected clonotype pairs (or specific single chains) may be added to a separate group or highlighted on the UMAP to identify the unique effector clusters for comparative scRNA-seq analyses (Figure 7). This facilitates the second analysis, differential gene expression, performed with selection of appropriate statistical comparison test using the dropdown menu in the “Groupings” section inclusive of *t*-test, Kruskal–Wallis, one-way analysis of variance (ANOVA), Wilcoxon rank sum test, MAST, Limma, paired *t*-test, paired ANOVA, or mean difference (see *RNA-seq plot viewer tutorials: performing differential gene expression*). The resulting differential analyses appear in a reordered “Genes” section, ordered according to rank and with representation of significance, relevant cell group (i.e., number 1 indicates this gene is more highly expressed in the first grouping), and comparative fold change in expression. More stringent significance can be applied by the user through adjusting *P*-value according to Holm, Hochberg, Hommel, Bonferroni, BH, BY, or false discovery rate, and genes lists reordered according to user preference on fold change or the number of cells expressing each gene (see *RNA-seq plot viewer tutorials: displaying number of cells with expression per gene*). Markers of interest for discovery studies may also be pulled to the top of the “Genes” domain via a simple name search, via the preset subsets tab, which contains predefined groups of markers, or by import from clipboard (see *RNA-seq plot viewer tutorials: genes: sorting, searching and selecting*).

**FIGURE 8:**
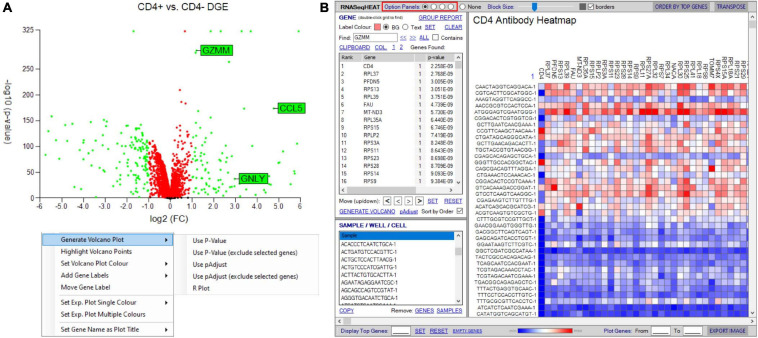
Tools to visualize differential gene expression. **(A)** Volcano plot for differential expression analysis between two groupings/samples. User-defined thresholds can be set for fold change and significance, visualized according to color (red and green represent genes above different preselected fold change (FC) and significance (*p*-value). Right-click menu provides options for addition and formatting of gene labels, plot coloring, and exclusion of selected genes. **(B)** RNASeqHEAT enables generation of a heatmap of selected genes as shown on horizontal axis and with selected samples on vertical axis. Option panel (highlighted in red) allows customization.

Initial differential gene analyses are best visualized by volcano plots incorporated into the “generate plots” function, separating differential RNA by both statistical inference and fold change (see *RNA-seq plot viewer tutorials: generating volcano plots*) (Figure 8A). If groupings have been chosen on select markers, these may be excluded from the plot as to prevent skewed axes and more accurately dissect data. Alternatively, traditional heatmap plots can be created using the “RNAseqHEAT” tool, of particular utility when more than two populations are being comparably assessed, again with user-adjustable heat scaling (Figure 8B).

**FIGURE 9:**
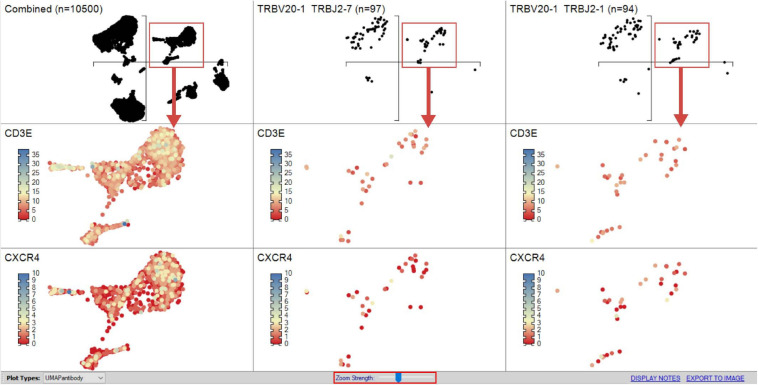
Heatmapping scRNA-seq gene expression on UMAP. Combined sample or subpopulation UMAPs can be compared on a single screen simultaneously providing an overview and more discrete dissection of sample. The “Zoom Strength” slider (highlighted red) enables focus on a particular region of interest directed by grouped analyses for heatmapping gene expression between samples as dictated by interest of prior differential gene expression analyses.

Expression of markers of interest may also be individually displayed as a heatmap directly onto the UMAP by selecting a specific marker in the gene list and accessing the one-click function tab at the bottom of panel labeled “heatmap” (see *RNA-seq plot viewer tutorials: coloring plots using heatmaps*) (Figure 9). Given a UMAP is a two-dimensional (2D) image of a 3D projection, respective arrow buttons plot those with highest expression at the front or back of the plot, respectively (see *RNA-seq plot viewer tutorials: setting the draw order of plot points*). Importantly, to aid in the visualization of multidimensional datasets, UMAPs may be aligned side-by-side to assess different zoomed regions of the total UMAP or the same populations from different samples and be similarly displayed as a heatmap with user-defined formatting using the right-hand-side menu. Currently, plots may be compared up to four columns wide and three rows deep on a single screen, but with additional tabs available with rapid toggle function such that unlimited parameters may be compared during a single analysis.

**FIGURE 10:**
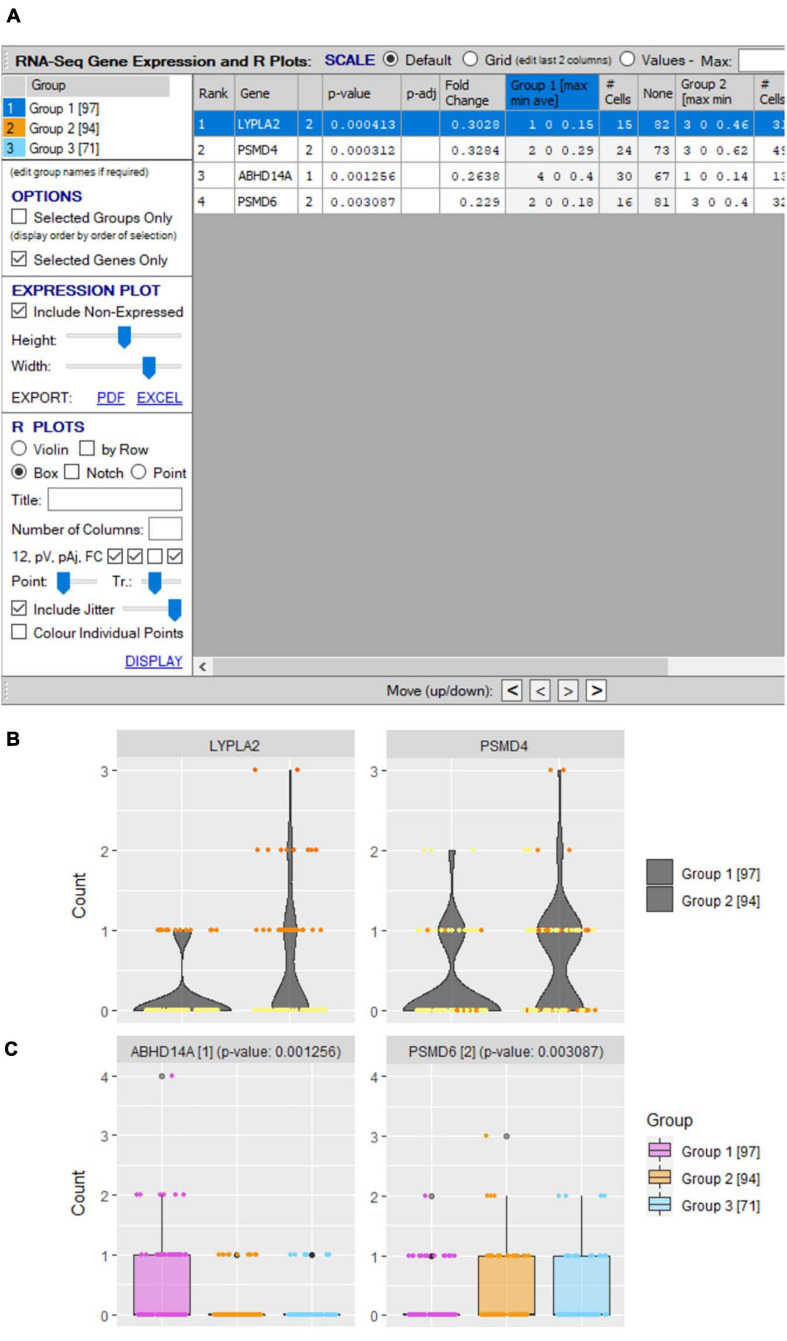
Differential gene expression plots. **(A)** “scRNA-seq gene expression and R plots” window allows user to define genes for inclusion in single/comparative **(B)** violin and **(C)** boxplots with optional inclusion of jitters and defined heatmap coloring of groups.

Significant differential expression does not necessarily infer predominant expression in a subset. If more finite expression level plots are required, this is performed in VGAS by simply highlighting the interest genes and selecting “Generate violin/box plots by gene(s)” from the right-click drop menu in the “Groupings” tab [see *RNA-seq plot viewer tutorials: generating gene plots* (Violin and Box plots etc.)]. Alternatively, a specific interest gene list in a specific order for presentation can be selected by using the “Select from clipboard” function. An initial window provides an overview of expression for each group showing the minimum, average, and maximum expression, along with the number of positive versus negative expressing cells for each selected gene (Figure 10A). Fully customizable violin or box plots are readily generated with displayed average expression, with added option to include jitters and/or heatmap colors to align directly with other figures [see *RNA-seq plot viewer tutorials: coloring gene plot (jitter) points, coloring gene plot (jitter) points using heatmaps*; Figures 10B,C].

### Utility of scCITE-Seq: Integrated Single-Cell Delineation of Surface Proteome

Transcriptome analyses provide highly complex cellular signatures of disease, revealing extensive heterogeneity among previously considered homogeneous populations (Bjorklund et al., 2016). However, RNA transcription profiles do not correlate completely with functional protein profiles, a more tangible measure of output functionality and more traditional diagnostic or therapeutic target. This divergence in comparative expression is a product of several processes including posttranscriptional processing, investigational error, and variable protein turnover, but importantly, there remains a direct RNA–protein concentration correlation, reported to be approximately 40% (Vogel and Marcotte, 2012).

Surface protein is traditionally assessed by indexed flow data, but this remains substantially limited by availability and spectral overlap of fluorescent tags and thus provides a far more restricted glimpse into the rich and diverse phenotypic landscape truly expressed on each and every cell. Thus, in recent years, unique tripartite DNA barcodes have been similarly generated and tagged to protein-specific antibodies unique to surface proteins of interest as to similarly sequence select surface protein expression on the same cell as scTCR–RNA-seq. Two separate modalities, CITE-seq and REAP-seq, respectively, were developed by teams at the New York Genome Center (NYGC) and Merck group (Peterson et al., 2017). While both are suitable for analysis in VGAS, we use scCITE-seq, reviewed in detail by the Stockieus laboratory at NYGC (Stoeckius et al., 2017), which is fully integrated with scTCR–RNA-seq. As with flow and mass cytometric methods, there remains a requirement to validate titrations for each antibody combined in a single panel, but we have found it straightforward to develop panels of more than 40 antibodies. We have optimized staining of different human samples, including skin, blood, adipose tissue, and blister fluid, using combinations of more than 45 oligo-tagged antibodies for the investigation of DHR and other diseases. This technology forms an integrated pipeline for holistic dissection of populations by single-cell transcriptomics and comparative surface protein markers and TCR.

### VGAS: scCITE-Seq Analyses and Visualization Tools

**FIGURE 11:**
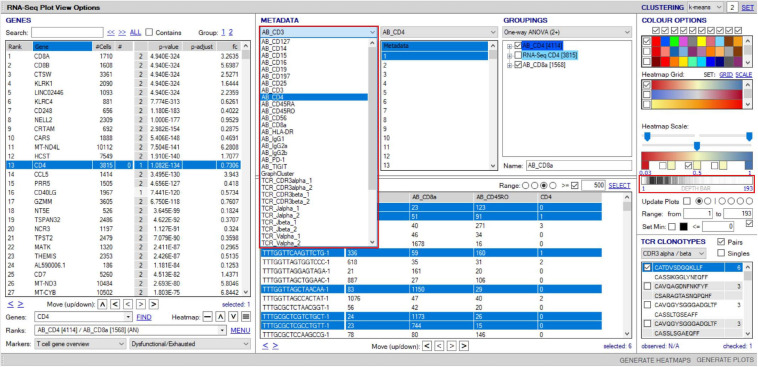
Overlay of surface protein expression (scCITE-seq) onto scTCR–RNA-seq analyses. Within the “RNA-seq plot viewer options” window, normalized CITE-seq data are included in the “Metadata” panel, accessible via the dropdown tab (highlighted red). All applications discussed for scRNA-seq analyses are applicable to protein with differential analyses between groups, selection into groupings or dissection via the “cell/sample metadata/domain. Depth bar (highlighted red) provides detail of protein expression density via a white-to-black scaling with darker areas representing most abundant expression.

**FIGURE 12:**
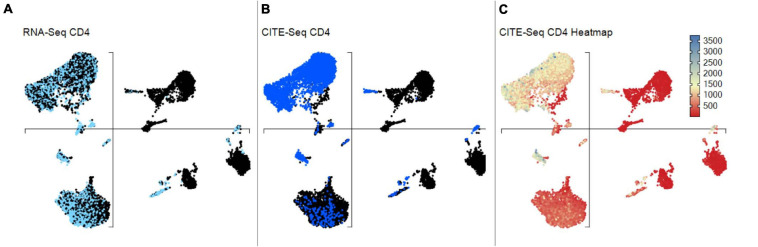
scCITEseq data visualization on scRNA-defined UMAP. Gene and corresponding surface protein expression can be compared across individual cells on the same unsupervised UMAP. **(A)** Cells highlighted with scRNA-seq defined positive CD4 expression. **(B)** Cells highlighted with surface CD4 expression using scCITE-seq antibody. **(C)** CD4 scCITE-seq antibody expression displayed as a heatmap onto scRNA-defined UMAP.

VGAS can incorporate scCITE-seq data, listing normalized count files for each antibody in the “Metadata” dropdown menu (Figure 11). Much of the analysis options described above for scRNA-seq are possible for CITE-seq including differential expression analyses with statistical inferences. Protein values can also be displayed as a heatmap directly onto the same UMAPs as scTCR–RNA-seq to delineate comparative RNA to protein expression (Figures 12A,B). If required, protein markers can be similarly explored and combined in the “Sample/cell metadata” columns with specific TCR pairs or RNA interest markers, generating a complete cell signature. Differential protein analyses are possible between groups with inference determined by varied statistical tests and heatmapping directly onto the same UMAPs for comparable expression plots with corresponding RNA (Figure 12C). Importantly, numerical positive expression gates must be reset for protein data with different expression ranges between markers. The right-click function allows the user to set coverage depth for a particular antibody detailing the numerical spread of expression, which appears in the depth bar (see *RNA-seq plot viewer tutorials: coloring plots using heatmaps*) (red highlight, Figure 11). This bar also provides detail of expression density via a white-to-black scaling, with darker areas representing the most abundant expression. This can be plotted next to the heatmap scale on UMAPs to provide comparative information on whether expression is high, mid, or low or even multimodal. This complements the visual given the 3D nature of the 2D UMAP such that some points may be hidden behind others. Heatmap scaling may then be adjusted by the user such to identify finite comparative expression even between two low-expressing populations. More familiar flow-type histograms are soon to be incorporated as an alternative gating and presentation strategy to dissect multimodal expression given the observed subtlety of variation on UMAP between similar but spatially distinct clusters. Our own analyses to date (data not shown) comparing flow-based and CITE-seq protein expression show distinct overlap with similar population representation, in line with that in the original pilot report from the Stockieus laboratory (Stoeckius et al., 2017).

### Brief Technical Overview and Performance Benchmark

The requirements and complexity of “big data” meant loading data files is typically slow, and subsequent analyses cumbersome. Thus, the VGAS development team, critically bridging software developers, bioinformaticians, laboratory scientists, and clinical researchers all with extensive experience of handling genomic data, has a major goal to develop and optimize performance solutions for individual data acquisition functions. Successful in this objective, VGAS incorporates non-standard and interlinked algorithms from different programming languages, most typically C sharp (C#), and provides highly efficient data structure solutions to enable everyday use on standard specification 8- to 16-GB laptops. Enhanced storage efficiency is critical for ease of file sharing and limits expense of long-term cold data storage whereby a 10-GB normalized gene file can be saved in an application specific (.VGASpv) plot view file and zipped to 350 Mb. Furthermore, modular framework of the application allows for inclusion of third-party tools and development of novel analysis modules without affecting functionality. Using R.Net to negate direct coding, VGAS utilizes widely available R-based packages, hidden to the user, adapting such to provide full customization of traditionally static R plots for scale, color, and resolution for rapid generation of publication-quality images available in all bitmap formats (tiff, png, jpeg) or as vector graphics. Importantly, VGAS has been programmed by an experienced software engineer through the entire software development “life cycle” using an integrated development environment, Microsoft Visual Studio, which provides comprehensive testing tools for validation. To provide a performance benchmarks, we utilized a standard eighth-generation i7 Windows-based laptop with m.2 SSD as now typically used in research institutions to load a 3-GB file representing counts for more than 23,000 genes and 120 metadata (integer, numeric, and factor) across 30,000 cells in less than 3 min. Active file upload used 4–5 GB, which was maintained even during subsequent active display of 12, albeit potentially unlimited, comparative UMAP plots. A crude sample split into two groups of 15,000 cells was then used to benchmark performance for differential gene expression analyses by one-way ANOVA, taking less than 3 min. To ensure speed of analyses, we recommend a laptop with 8-GB RAM and i5 processor or equivalent as the minimum hardware specification.

Prior to investigator release, raw sequencing data are run through a standard data normalization and quality-assurance pipeline by our on-site bioinformatician. Importantly, VGAS is independent of this standard pipeline, which uses open-access tools, and may load data processed by any best-practice approach. For droplet-based (10×) assays, Cell Ranger is used to align data from 10X-5′sc-RNA-TCR-CITE-seq, with processing of antibody-derived reads by Seurat^3^ to analyze the multimodal data and cluster cells based on both RNA and protein expression. scRNA-seq measurements are normalized using SCTransform and scale-factor transform method. scCITE-seq measurements are normalized using centered-log ratio transformation in Seurat. Cells with fewer than 200 genes and more than 10% mitochondrial content are typically removed, but alternate cutoffs may be defined by individual investigators. Furthermore, genes with more than 0 counts in fewer than three cells are also typically removed. Initial first-pass analyses are performed using a combination of most current versions of Seurat for unsupervised clustering and SingleR for cell-type prediction. After normalization, data input for VGAS is a comma-separated (.csv) normalized expression file and tab-separated metadata text file (.txt), with the sample identifier as the first column and a second column to display metadata as factor, integer, or numeric value. This easily modifiable format enables additional metadata to be included if not known at time of initial analysis. We also provide a UMAP/t-SNE.csv file to view x, y coordinates; however, data are also released as a merged VGAS plot view (VGAS.pv) master file, which encompasses all three individual files (normalized.csv, UMAP/t-SNE.csv, metadata.txt) for simple one-click file open during software trial. All four test data files are available to view input by download from our website^4^ (“VGAS RNA-Seq Plot Viewer files” provides download link to all four VGAS files (metdata.txt, normaliation.csv, UMAP.csv, VGAS.pv).

### Summary and Future Development

Single-cell sequencing technologies are revolutionizing our ability to understand complex cellular systems, providing unparalleled ability to dissect the previously undefinable finite cellular discrepancies that delineate disease phenotypes, patient outcomes, and treatments. Moreover, because only 1,000–2,000 cells are thought to be required for sufficient *de novo* population dissection of any heterogeneous sample (Giladi and Amit, 2018), it is feasible to explore even limited clinical samples from relevant reaction sites. Collaborative effort to map the entire human body by cell in the Human Cell Atlas provides a critical reference resource to facilitate interpretation of the results (Regev et al., 2017). For T-cell–mediated disease such as DHRs, the integration of multifocal scTCR–RNA–CITE-seq provides a pipeline to identify actively responding tissue-based effectors driving destructive outcome, first identifying dominantly expanded or newly recruited TCR, before determination of unbiased transcriptome and select proteome signature to delineate disease- and tissue-specific biomarkers. This is possible not only for critical T-cell effectors, but also all accessory populations, enabling more complete understanding of the entire cellular microenvironment. These single-cell technologies require investigators to be able to manage and analyze their own big data. Investigators are at risk of losing the understanding of their own data if they are completely dependent on bioinformatics experts with the coding expertise required for suitable data handling, quantification, and analysis. VGAS is specifically designed to enable researchers to be increasingly self-sufficient. One limitation is that the program has to be administrator-distributed and linked to the highly specialist sequencing pipelines available at a few specialist genomics facilities worldwide. Critically, such sequencing is a core service provided to external laboratories by our center with VGAS made freely available for download to investigators. Access is also provided to researchers who run their own sequencing; however, there is a requirement for VGAS access that initial bioinformatics normalization must be performed in collaboration. However, prospective users can request for a 2-week trial of VGAS by contacting software@iiid.murdoch.edu.au.

VGAS is strategically a non-web-based, Windows-aligned application to minimize the effect of variable download speed, allow analysis on the go, provide highest resolution images for presentation, and, moreover, provide means to secure confidential data to internal servers. Background coding has been meticulously performance-optimized for different functions to provide timely analyses that run on standard laptops without need for high-end hardware and servers. With the fundamental vision to provide researchers with intelligible means to access and interrogate single-cell data and develop publication-ready figures, a key ongoing principle remains user-directed development. Next versions under construction are to include flow-type histogram-based gating for alternative dissection of multimodal RNA and protein expression and trajectory analyses of pseudo time. Pseudo time, or “pseudotemporal reconstruction,” an inference of time through the shortest transcriptomic path between all linked populations, provides insight into both the transcriptome-predictive before and after precursors and exhausted counterparts of identified effector populations. This enables conversion of what is a static snapshot of cellular transcriptome in time into a continuum of cellular development given the breadth of developmental stages for each cell type, based on subtle discrepancies in similar gene expression (Haghverdi et al., 2016). Importantly, interest transitions may be verified by transposase-accessible chromatin sequencing (ATAC-seq) to inform upon the plasticity from epigenetic modifications toward a specific signature within a lineage as described by Gury-BenAri for helper-like innate lymphoid cells in healthy mouse intestine (Gury-BenAri et al., 2016). Thus, from a single sample, a longitudinal view of cellular response may be obtained. These additional analyses performed by our bioinformatics team will not alter the input data, but rather extend the existing metadata matrix, for example, by providing pseudotime clustered populations with n = populations set by user. Further information on our dedicated webpage (see text footnote 4) will guide prospective users through the plethora of sequencing technologies and associated analyses provided by VGAS.

## Data Availability Statement

The original contributions presented in the study are included in the article/supplementary material, further inquiries can be directed to the corresponding author/s.

## Ethics Statement

The studies involving human participants were reviewed and approved by Murdoch HREC. Written informed consent for participation was not required for this study in accordance with the national legislation and the institutional requirements.

## Author Contributions

SM, EP, SL, AC, and MW conceptualized VGAS. SL, RR, and DC contributed to technical design. RH, PD, and AG contributed to analysis. RH, PD, AG, and RR wrote the manuscript. AC, SL, SM, YL, AP, AG, and EP reviewed the manuscript. All authors have approved the submitted manuscript.

## Conflict of Interest

VGAS is proprietary software owned by Murdoch University who employ many of the lead authors. The authors declare that the research was conducted in the absence of any commercial or financial relationships that could be construed as a potential conflict of interest.

## Footnotes

1 https://www.iiid.com.au/software/vgas/tcranalysis/

2 https://www.iiid.com.au/software/vgas/vgas-rnaseqplotviewer

3 https://satijalab.org/seurat/

4 https://www.iiid.com.au/software/vgas
